# Everybody's Page

**Published:** 1888-06-16

**Authors:** 


### EVERYBODY'S PAGE.

LEPROSY is said by M. Besnier to be spreading rapidly instead of dying out, as has been commonly supposed.

PROPER clothing is to be looked upon as an equivalent for food. It favours longevity by diminishing expenditure.

NEVER take a house of which the daylight is so deficient that artificial illumination will be required during the day.

HAIRDRESSERS have a mortality half as great again as that of the agricultural labourer ; publicans die almost as rapidly as cabmen, and at just twice the rate of the clergy.

PERSONS who have been for a long time exposed to the action of lead, such as workers in white lead factories, plumbers, and painters, sometimes suffer from disease of the kidneys produced by the irritation of the lead which they (the kidneys) discharge from the body.

THE nearer people live to each other the shorter their lives are. Statistics show that where the density is 166 persons to the square mile, the death-rate is 17 per thousand ; where it is 1,718, the death-rate is 25 ; and where the population is 12,357 per square mile, the death-rate is 38 per thousand.

PROFESSOR VOISIN claims to have cured an inveterate liar of his evil habit by mesmerism. Professor Voisin is not wanted by dealers in patent medicines. Against this marvellous success may be set the fact that a physician in Carlsruhe hypnotised a young man, who remained in the trance state for eighteen hours, and then became maniacal.

"ACROMEGOLY" is the title of a curious disease in which the feet, hands, and face are affected—enlarged and distorted almost beyond recognition. Even acromegoly, however, has its compensations. One patient suffering from it is said to be earning a respectable income in a travelling show. In perfect health he might have been one of the unemployed.

NEARLY twenty years ago Dr. Wilks directed attention to the fact that a transverse furrow appeared on the nails of the hand after a serious illness. Medical literature has since then contained a few references to the subject. He again brought the subject before the Pathological Society at its meeting on March 20th, and related a remarkable case. In that case the furrow was caused by three days' sea-sickness.

FRITSCH and Hitzig in Germany, and Professor Ferrier in this country, have found that the stimulation of various circumscribed areas on the surface of the convolutions of the brain (which till lately was believed to be insensible to irritation) is followed uniformly by movements of particular limbs. For instance, if a mild current of electricity is applied to one spot, the leg is moved ; if to another, the eyes ; if to a third, the neck, muscles of the face, and so on.

THE law is so kind as to make minute regulations on the subject of the sailor's grog. Every ship must carry a sufficient quantity of lime or lemon-juice as an anti-scorbutic, containing fifteen per cent. of proper or palatable proof spirits. Proper and palatable spirits are defined by Statute to be sound rum of a specific gravity from ·074 to ·920, or sound brandy of a specific gravity not less than ·920. It is amusing, however, to observe that the Board of Trade actually takes the trouble of mixing the grog for the seamen. They recommend that one ounce of the lime-juice should be mixed with one ounce of sugar and at least half-a-pint of water and should be served out in time for dinner.

THE risk of chills, which in the ordinary routine of life cannot altogether be avoided, may be diminished by the systematic use of the cold bath.

IN many occupations, as knife or needle grinders, potters, stonemasons, &c., the air is impregnated with a fine dust. This gets breathed, carried into th e lungs, and mechanically irritates them, causing consumption.

SOME anthropologists are inclined to believe that the earliest men who peopled the globe were red-haired, because amongst all human races individuals have been observed whose hair more or less approaches to this tint.

FOR those who have much physical work to do, two articles in common use ought to be very carefully indulged in—tobacco and alcohol, inasmuch as they have a decidedly weakening effect on the heart.

THE main danger to be apprehended from indulgence in athletics is their being undertaken when the individual is not in "condition," or when he is too old. A game like football should be played regularly, if played at all, and there should be a life calendar of games for the different ages of man, beginning perhaps with football, and running on with cricket and tennis, to bowls, golf, and archery.

IN Canton in 1858 the number of Chinese barbers is said to have exceeded 7,000. Most of the Fijian chiefs employ a hairdresser, whose whole duty is centred in the care of his master's hair. In the Latooka tribe it takes seven or eight years to complete the head toilet. As a general rule, in uncivilised countries it is the men who thus adorn themselves. In Marquesas Islands, indeed, the husband sometimes sacrifices his wife's hair towards his own adornment. He shaves her head, and appropriates her hair for his own crown.

AN averaged-size man, engaged in very moderate work, breathes—that is, inspires and expires—about 1,000,000 cubic inches of air every 24 hours—every day. This amount is more than 500 cubic feet, and more than 3,000 gallons of air. Were we required to purchase this quantity of air food, each of us would have to order a case or box full of air 10 feet long, 10 feet broad, and 5 feet high, or if we chose to have it in quart bottles, we should have delivered to us more than 18,000 quart bottles per day.

A BEARD is by no means universal amongst men. Thus the Siamese, Malays, Chinese, and Japanese are beardless, and so also are the native Americans. As a rule these races express strong objections to beards. The Japanese ladies consider them "very ugly," and the North American Indians consider them "very vulgar." Upon this principle the latter carefully eradicate with pincers every straggling hair which may from time to time appear on the face. The Indians of Paraguay carry this practice so far that they in addition pluck out both eyebrows and eyelashes. As a reason for doing this they state that they do not wish to look "like horses."

A CURIOUS application has been made of the properties of lanoline in the treatment of that trouble of fading youth—wrinkles. It does not matter how juvenile one's mind is, how ready one is to take part in the delightful though somewhat fatiguing and frivolous sports of youth, the intrusive crow's foot betrays all. Lanoline, however, when applied to the skin, and well rubbed in, acts as a nutrient to the subjacent tissues, with the effect of smoothing out the folds produced by the attenuation of these structures incidental to age. Several ladies who have tried the lanoline treatment are said to be delighted with the result.

